# Properties of Styrene-Maleic Anhydride Copolymer Compatibilized Polyamide 66/Poly (Phenylene Ether) Blends: Effect of Blend Ratio and Compatibilizer Content

**DOI:** 10.3390/ma13153400

**Published:** 2020-07-31

**Authors:** Alper Aksit, Nico Geis, Merve Aksit, Volker Altstädt

**Affiliations:** 1 Department of Polymer Engineering, University of Bayreuth, Universitätsstraße 30, 95447 Bayreuth, Germany; alper.aksit@uni-bayreuth.de (A.A.); nico.geis@uni-bayreuth.de (N.G.); merve.aksit@uni-bayreuth.de (M.A.); 2 Bavarian Polymer Institute and Bayreuth Institute of Macromolecular Research, University of Bayreuth, Universitätsstraße 30, 95447 Bayreuth, Germany

**Keywords:** PA66, PPE, blend ratio, compatibilization, SMA, morphology, mechanical properties

## Abstract

Two different blend ratios of polyamide 66 (PA66) and poly (2,6-dimethyl-1,4-phenylene ether) (PPE) (60/40 and 40/60 *w*/*w*) were produced via melt mixing. A styrene–maleic anhydride copolymer (SMA) was utilized at various contents from 2.5–15 wt% to compatibilize the immiscible blend system. The influence of SMA content and blend ratio was investigated based on (thermo-) mechanical and morphological properties of the PA66/PPE blends. Correlations between the interaction of SMA with the blend partners were established. For 60/40 blends, a droplet-sea morphology was visualized by transmission electron microscopy, wherein no major changes were seen upon SMA addition. In the case of 40/60 blends, strong coalescence was found in the binary blend. Up to 5 wt% SMA, the coalescence was inhibited by the interfacial activity of SMA, whereas 10 wt% SMA initiated a disperse-to-co-continuous transition, which was completed at 15 wt% SMA. An enhancement of tensile properties was achieved for all blends possessing SMA, where the maximum concentration of 15 wt% resulted in the highest elongation at break and tensile strength values. The relative improvement of the tensile properties was higher with the PPE-rich blend (40/60) which was attributed to a partial emulsification of the PPE phases forming a bimodal PPE domain size distribution with nano-droplets in the range of 60–160 nm.

## 1. Introduction

Melt blending of different types of thermoplastic polymers is a useful method to develop materials with desired properties for a variety of applications from automotive to packaging sector [1,2,3]. Since almost all polymer blend combinations reveal immiscible morphology due to macrophase separation, the mechanical properties of the materials deteriorate [4,5,6,7,8]. As phase separation is inevitable, a lot of effort has been done to control the apparent morphologies by various compatibilization methods, such as multi-step reactive extrusion [9,10,11,12,13,14,15] and the addition of inorganic or hybrid nanoparticles [16,17,18,19,20,21,22] and copolymers with various functionalities [23,24,25,26,27,28].

Polyamide (PA)/ poly (2,6-dimethyl-1,4-phenylene ether) (PPE) blends have gained great interest in engineering applications, i.e., electronics, marine and automotive. The strategy of blending yields in a material with a low water absorption, heat resistance, dimensional stability, and resistance to many chemicals. Similar to other blends, PA/PPE blends are immiscible and lack of stability under mechanical stress [29]. To overcome this drawback, mainly copolymer type compatibilizers are used, following a physicochemical mechanism. On the one hand, physical interactions expressed by chain entanglements between the copolymer backbone and the PPE are observed [30,31]. On the other side, moieties of the copolymer undergo covalent bonding to the PA, either linking to the carboxylic or amino end-groups [32,33,34]. Styrene-maleic anhydride copolymer (SMA) is one of the physicochemical compatibilizers recently applied for PA/PPE blends to strengthen the interfacial interaction, morphology and mechanical performance [35,36,37,38]. A broad variation of different maleic anhydride (MA) concentrations allow for the tuning of interactions of the SMA with the blend partners. With a lower MA concentration, good miscibility in PPE is achieved, though weaker interactions with PA are expected. Higher MA concentrations result in a non-miscible mixture of PPE and SMA and strong reaction with PA. The full miscibility of SMA in PPE is reported in the literature up to 8 wt% MA [39,40].

The influence of MA concentration on polyamide 6 (PA6)/PPE blend properties was studied in literature, where a MA concentration of 8 wt% provided better compatibilization compared to a SMA with 2 wt% MA [41]. A SMA with 8 wt% MA used by Chiang and Chang [37] showed the effect of SMA content and the blend ratio on the mechanical properties of PA6/PPE/SMA ternary blends. For a fixed PA6/PPE ratio of 50/50, an improvement in tensile and impact properties, reaching a maximum at 10 wt% SMA content, was reported. Besides the 50/50 blend ratio, the researchers varied the blend ratios as 70/30 and 30/70 PA6/PPE. In all cases, 10 wt% SMA resulted in the highest values for tensile and impact strength. It was stated that the performance of the blends increases with larger amounts of PPE. In relative numbers, the tensile strength increased by 50%, 65%, and 118% for 70/30, 50/50, and 30/70 PA6/PPE, respectively in comparison with the blends with and without 10 wt% SMA. A structure-property relationship between the apparent morphology, the mechanical performance and the fracture mechanisms however is not given in respect to tensile testing. When discussing blend properties, impact tests have mostly been applied and discussed in detail [30,34], yet not all blends are exposed to impact stresses in regard to their application. We believe that tensile testing is a more versatile method to investigate.

Recently, scientific focus is shifted towards using polyamide 66 (PA66) instead of PA6, due to its superior thermal and mechanical properties and slightly lower humidity/water absorption (Table A1 in Appendix A). Similar to PA6/PPE, PA66/PPE blends also require compatibilization to ensure a sufficient interfacial adhesion between the PA66 and PPE phases. Compatibilization is mainly performed by PPE-graft-MA [42,43,44], organo-montmorillonite [45] and SMA/(styrene-ethylene-butylene-styrene copolymer)-graft-MA dual compatibilizers [46]. In our previous study [47], we established structure-property-relationships of SMA compatibilized PA66/PPE blends for the first time. We investigated the effect of two different SMA copolymers (8 wt% and 25 wt% MA concentration) on the morphological and mechanical properties. It was found that for PA66/PPE blends (50/50 *w*/*w*), a lower MA concentration (8 wt%) results in better mechanical properties compared to the SMA with 25 wt% MA. The higher efficiency of 8 wt% MA concentration was attributed to a high interfacial activity, provided by the miscibility with PPE. In contrast, SMA with 25 wt% was found to be immiscible in PPE, thus no improvement of the blend interface was observed. With a weak interfacial adhesion, deteriorated mechanical properties were obtained. For the first time, we postulated the nano-emulsion formation of PPE within a PA66 polymer matrix, initiated by SMA. Here, we were able to distinguish between unswollen and PPE-swollen PA66-g-SMA micelles, given by their difference in size. Nonetheless, the influence of PPE to SMA ratio on the swelling behavior of the micelles is not cleared satisfyingly.

Besides the variation of the SMA type and content, the blend ratio of PA66/PPE also influences the interaction intensity of SMA with any of the blend partners. Higher amounts of PA66 offer more reactive amine moieties, thus stronger grafting of SMA is expected. In contrast, a higher PPE content provides shorter diffusion paths for SMA to reach the PPE domains. The influence of PA66/PPE blend ratio was mentioned in two independent studies [44,46], each comparing two different PA66/PPE blend ratios, 45/55 with 75/25 [46] and 50/50 with 70/30 [44]. Both studies agree on superior tensile and impact properties for higher PA66 contents. A more comprehensive comparison of PA6/PPE ratio was proposed by [43], where five different blend ratios were investigated from 70/30 to 30/70. It is noteworthy that a previously modified PPE (PPE-g-MA) was used in all cases. The workgroup stated that the tensile strength is lowest with the highest PA66 content of 70 wt%, which does not coincide with the findings from [44,46]. Additionally, they achieved maximum tensile strength values with blend ratios of 60/40, 50/50, and 40/60. However, a deep understanding and a systematic correlation of the mechanical properties to morphological characteristics do not exist.

To the best of our knowledge, a profound study regarding the effect of PA66/PPE blend ratio is completely missing in the literature, where SMA is used as a compatibilizing agent. Consequently, we strive to gain deeper insights on the influence of blend ratio (60/40 and 40/60 *w*/*w*) and SMA content (0–15 wt%) on PA66/PPE blends. Correlations between blend morphology and the corresponding tensile properties and the apparent fracture mechanisms are established. In addition, the swelling behavior of PA66-g-SMA micelles, depending on the blend ratio and SMA content, are highlighted.

## 2. Materials and Methods

### 2.1. Polymers

Commercially available neat PPE powder, compounding grade PA66 and SMA were processed as provided. Important product properties are given in Table 1. Size-exclusion chromatography (SEC) was performed to measure the molecular weight (M_w_) and the polydispersity. The chromatograph included four single-dose vial gel columns (filler size = 5 µm) with a given porosity between 102 to 105 Å (Polymer Standards Service GmbH, Mainz, Germany) and a non-selective refractive index detector (Shodex, Techlab, Tokyo, Japan). Chloroform (CHCl_3_) and hexafluoroisopropanol (HFIP) were used as solvents for PPE and PA66, respectively. A flow rate of 1.0 mL/min was set for the eluent. Poly (methyl methacrylate) and low-polydispersity polystyrene (PS) were used for calibration of PA66 and PPE respectively.

### 2.2. Blend Processing

As PA66 is sensitive to hydrolysis, the granules were thoroughly dried at 80 °C and 16 h by using a dry-air granulate dryer (TLE 100, Gerco Technik GmbH, Enningerloh, Germany), having a residual water content lower than 950 ppm (0.0950 wt%). Dosing of the granular materials was performed by a gravimetric feeder equipped with a single-screw (Coperion K-Tron K2-ML-T35-QC, Stuttgart, Germany). The PPE powder was metered with an interlocking twin-screw used in a gravimetric feeder from Coperion (K-Tron K-SFS, Stuttgart, Germany). All materials were metered in the main feeding zone at a constant total feeding rate of 10 kg/h for all blending steps. Compounding was performed with a rotation speed of 300 rpm and 270 °C mean barrel temperature, using a co-rotating twin-screw extruder (ZSK 26 MCC, Coperion GmbH, Stuttgart, Germany). Strand-pelletizing was applied after quenching the strand in a water bath. A two-step processing was chosen for the PA66/PPE/SMA blends, whereas one-step compounding was carried out for the PA66/PPE binary blends. For the ternary blends, PA66 and SMA were reactively compounded first. The PA66/PPE blend ratios were fixed at 40/60 and 60/40 (*w*/*w*). For both blend ratios, the SMA amount was varied starting from 2.5, 5, 7.5, 10, to 15 wt%.

Specimen preparation for mechanical characterizations was conducted via injection molding (Arburg Allrounder 470H 1000-170, Arburg GmbH, Loßburg, Germany) of the dried granules. All parameters were kept constant for both, the binary and ternary blends, where the mold and nozzle temperature were set at 100 °C and 290 °C, respectively. A closing time of 20 s was applied to assure sufficient cooling prior to ejection of the specimen.

### 2.3. Differential Scanning Calorimetry (DSC)

Glass transition temperatures (T_g_) of the neat PPE, SMA, and binary blends of PPE and SMA were determined by a Mettler Toledo DSC 1 (Mettler Toledo, Giessen, Germany) device. A heating–cooling–heating cycle was applied from 25 to 300 °C under nitrogen atmosphere and a scanning rate of 10 K min^−1^. To calculate the T_g_ in accordance to ISO 11357-2 [49], the second heating cycles were considered. Herein, the center temperatures were determined as described in the named standard. Two measurements for each material were applied to increase the precision of the results.

### 2.4. Dynamic Mechanical Analysis (DMA)

Thermo-mechanical properties were characterized using a Gabo Eplexor 500N (NETZSCH-Gerätebau GmbH; Selb, Germany) DMA. While heating from 25–250 °C (2 K/min) an oscillatory stress of 2.5 MPa and a frequency of 1 Hz was utilized in tensile mode. For interpreting the results, tan δ signals were considered, where occurring glass transitions appear as peaks. Each material was measured three-times in order to minimize experimental errors. The T_g_ values were reported as an average of three tan δ peak values. Storage moduli (E’) data are plotted against temperature to support the discussion.

### 2.5. Rheological Characterization

A stress-controlled dynamic-mechanical rheometer (RDA III, Rheometrics Scientific, Piscataway, NJ, USA) was used to evaluate the rheological properties. A plate-plate setup with 25 mm diameter and a gap of 1 mm was selected for the measurements under nitrogen atmosphere. A measurement temperature of 270 °C and a deformation rate of 10% were kept constant for the applied frequency sweep from 0.1–500 rad s^−1^. Triple determination was utilized for all blends to assure the reproducibility. In addition, storage moduli (G’) data is plotted to give further insights regarding the molecular structure of the blends.

### 2.6. Morphological Characterization

For morphological analysis, a transmission electron microscopy (TEM) Zeiss EM922 OMEGA (Zeiss NTS GmbH, Oberkochen, Germany) was used at an acceleration voltage of 200 kV. Dog-bone specimens were used to prepare ultrathin sections (~60 nm) using an ultra-microtome (Leica EM UC7, Leica Microsystems GmbH, Wetzlar, Germany). For contrast enhancement ruthenium tetroxide was applied for 15 min, giving the PPE phases a darker appearance. Domain size distributions were calculated from 100 individual domains. With the help of the software “ImageJ” the longest diameter of each domain was measured. A size filter was applied prior to statistical evaluation, wherein only domain sizes up to 3000 nm were considered. Domains larger than 3000 nm are usually generated by coalescence, indicated by strongly irregular shapes. As we could not see structures smaller than 200 nm for the binary blends, another filter was set for the domain counting of the nano-sized structures exhibited in the ternary blends, cutting-off sizes larger 200 nm.

Field-emission scanning electron microscopy (FESEM) Zeiss LEO 1530 (Zeiss NTS GmbH, Oberkochen, Germany) was used for the characterization of the fracture surfaces at an acceleration voltage of 3 kV. Representative tensile bars, with values closest to the average were sputtered with a platinum layer (max. 2 nm thickness) in advance.

### 2.7. Mechanical Characterization

Young’s modulus, tensile strength, and elongation at break data were obtained by using a universal testing machine (Zwick Z020, ZwickRoell GmbH & Co. KG, Ulm, Germany) equipped with an extensometer. All measurements were performed by using specimen (Type 1A) as specified in ISO 527-2 [50]. Prior to testing, all specimens were dried overnight at 80 °C in vacuo and vacuum sealed to exclude humidity.

## 3. Results and Discussion

### 3.1. Interactions of SMA with the Blend Partners

**Figure 1:**
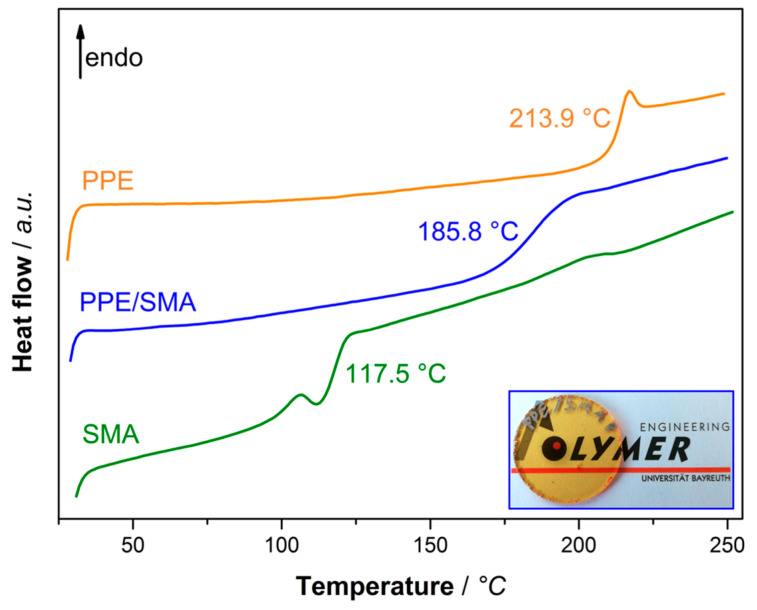
DSC thermograph of poly (2,6-dimethyl-1,4-phenylene ether) (PPE) (orange), Styrene-maleic anhydride copolymer (SMA) (green) and PPE/SMA blend (blue) at the blend ratio of 78/22. Hot-pressed disc of PPE/SMA blend for illustration of miscibility (right corner).

In order to gain information about the specific interactions, such as the miscibility of two or more components, methods like DSC, DMA, and electron microscopy are commonly used. Upon mixing of two miscible polymers, the individual T_g_ signals merge to a single response in DSC and DMA analysis [4]. For a non-miscible binary blend, each of the two T_g_-signals mostly remain unchanged after processing. In Figure 1, the DSC thermographs of PPE (orange), SMA (green), and PPE/SMA binary blend (78/22 *w*/*w*) (blue) are given.

As both polymers are amorphous, only a T_g_ signal (no melting temperature) appears at 117.5 and 213.9 °C for SMA and PPE (Figure 1, green and orange), respectively. When PPE and SMA are mixed at a given ratio, the T_g_ signals coincide at 185.8 °C (Figure 1, blue). For estimation of the mixed-T_g_, Fox equation (Equation (1)) will be used, where T_g_ (blend) is the T_g_ of the binary blend of PPE/SMA and *w* is the mass fraction of each blend partner, namely PPE and SMA.

1/T_g_ (blend) = *w* (PPE)/T_g_ (PPE) + *w* (SMA)/T_g_ (SMA) (1)

Equation (1) fits the experimental data quite well, as the calculated T_g_ equals to 186.4 °C. This approves the applicability of the linear mixing rule for miscible polymers. A hot-pressed rheology disc of the PPE/SMA blend validates the DSC result (blue curve) as a transparent blend is obtained upon mixing (Figure 1, bottom right).

**Figure 2:**
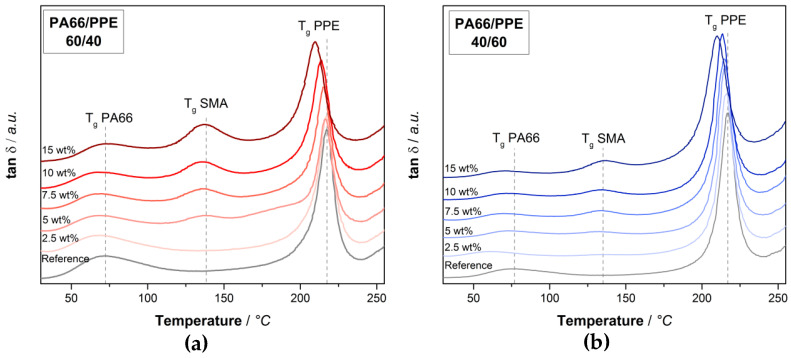
Tan δ plots polyamide 66 (PA66)/PPE/SMA ternary blends with (**a**) 60/40 blend ratio, (**b**) 40/60 blend ratio at various SMA contents.

Since the SMA is first blended with PA66 in case of the ternary blends, one can expect a different interaction of the SMA with PPE. The tan δ responses for both blend ratios at varying SMA contents are depicted in Figure 2.

Looking at the blend ratio of 60/40 (Figure 2a) a signal of SMA at around 135 °C is visible for 5 wt% and higher SMA content. Even though SMA is completely miscible with PPE, a large fraction of the SMA is not located within the PPE phase. Since PA66 and SMA form covalent bonds during the first extrusion step, the SMA chains are constraint, thus not allowing a complete diffusion into the PPE phase. With increasing amount of SMA, the peaks get more pronounced, indicating that the amount of SMA in the PA66 phase or at the PA66/PPE interface increases. Simultaneously, a shift of the PPE peaks is visible with the introduction of SMA, where a maximum shift is achieved at the highest SMA content of 15 wt%. A certain content of SMA is able to diffuse into the PPE phase, thus decreasing its T_g_ by 7 °C with 15 wt% SMA. In the case of the 40/60 blends (Figure 2b), similar observations can be made, yet the PA66 and SMA signals appear less pronounced, whereas the intensity of the PPE peaks is larger. Interestingly, a shift in blend ratio does not affect the degree of peak shifts for any of the components within the ternary blend. The peaks of PPE and SMA are summarized in Table 2, where the neat blends and blends with 15 wt% SMA for both blend ratios are considered.

For both blend ratios, the PPE peaks reveal a shift in the same order of magnitude when 15 wt% SMA is added. These results are in good agreement with the findings in [37] where a shift in the T_g_ of PPE is evident. Starting from 217 °C a reduction to 210 °C is observed for both blend ratios. Since the 40/60 binary blend contains 50% more PPE compared to the 60/40 binary blend, the interaction between SMA and PPE becomes stronger with increasing PPE content. To understand the effect of SMA on the thermomechanical stability, the storage moduli are given in Figure A2 in the same manner as Figure 2. For both blend ratios, the SMA leads to a strong loss in modulus at approx. 130 °C as a result of softening of SMA. For 60/40 blends, the SMA gives rise to a decreased modulus at ambient temperatures, whereas a contrary behavior is observed for the 40/60 blends. PPE has a lower modulus than dry PA66 and SMA, thus the addition of SMA to the PPE-rich blends will increase the modulus. All observed effects are enhanced with an increasing amount of SMA.

**Figure 3:**
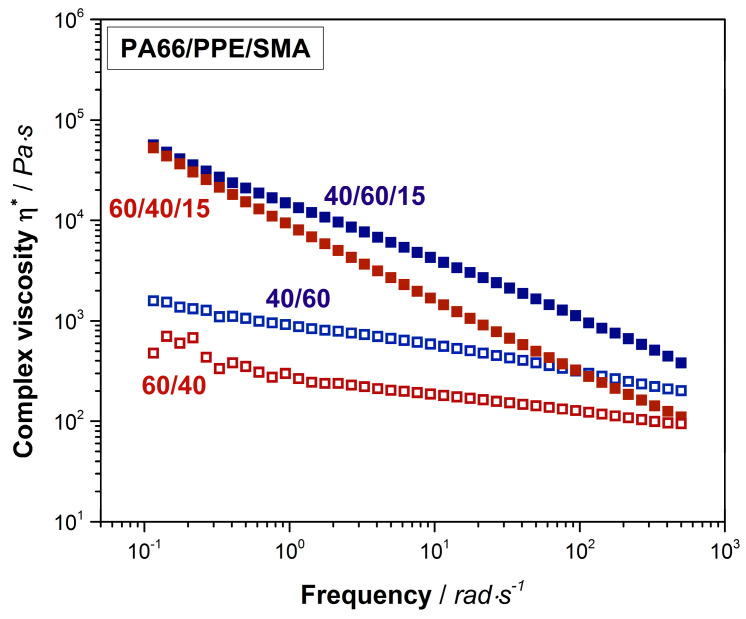
Shear rheological results of 60/40 (red hollow rectangular) and 40/60 (blue hollow rectangular) neat blends and their equivalents with 15 wt% SMA (red and blue rectangular).

Finally, interactions between SMA and PA66 are analyzed by shear rheological experiments as depicted in Figure 3.

**Figure A1:**
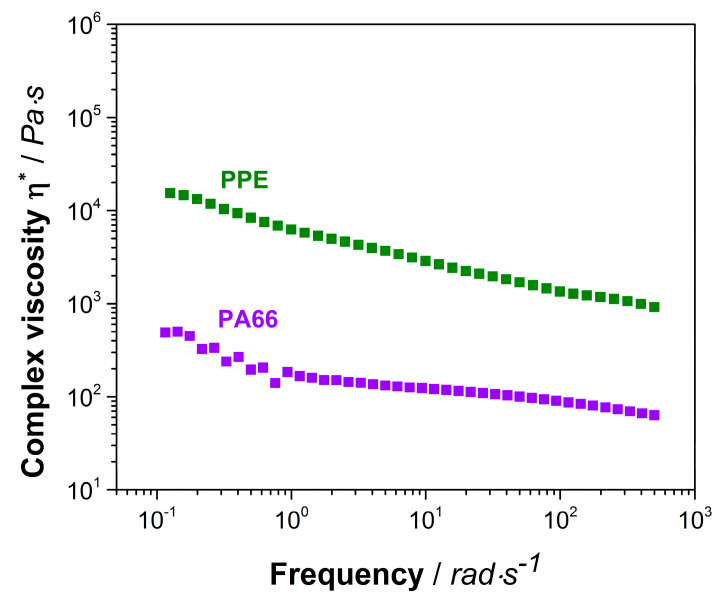
Complex viscosity of neat PA66 (violet rectangular) and neat PPE (green rectangular).

**Figure A2:**
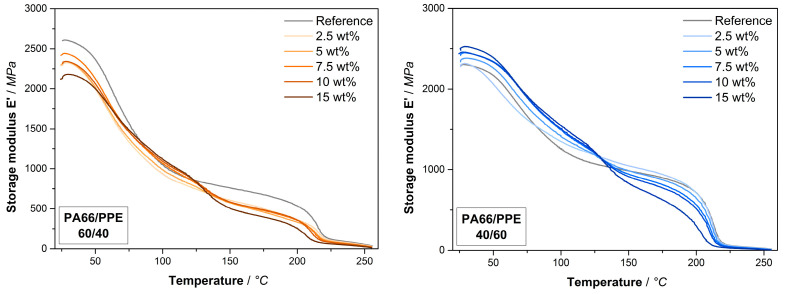
Storage modulus data of the PA66/PPE/SMA ternary blends with 60/40 blend ratio (**left**) and 40/60 blend ratio (**right**) at various SMA contents.

**Figure A3:**
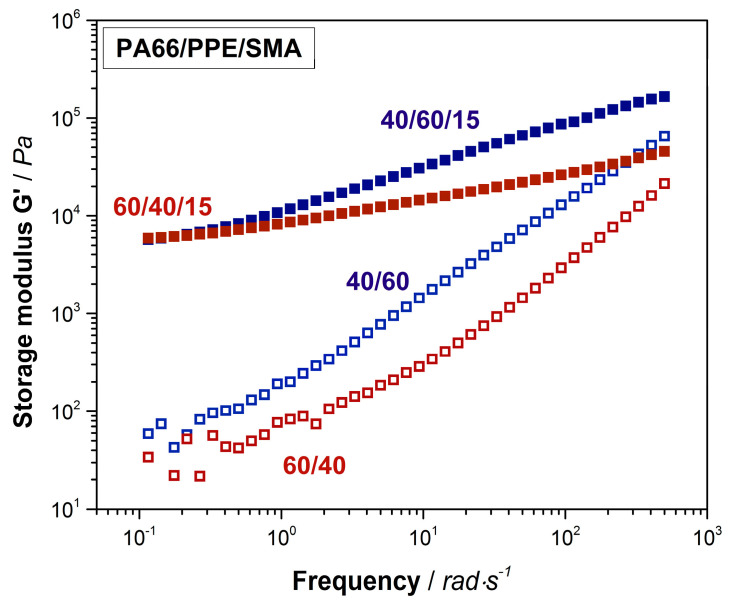
Shear rheological results of 60/40 (red hollow rectangular) and 40/60 (blue hollow rectangular) neat blends and their equivalents with 15 wt% SMA (red and blue rectangular).

PPE as an amorphous polymer, displays a relatively high viscosity compared to the one of semi-crystalline PA66 (Figure A1). Due to this fact, the complex viscosity of the 40/60 PA66/PPE blend is higher than the 60/40 blend over the complete frequency range. The trend of both curves conforms to the ones of typical linear thermoplastic polymers, however at lower frequencies no clear plateau is seen. This might be explained by reactions between PA66 and PPE during blending, leading to block-copolymer formation. When 15 wt% SMA is added, a strong linearity for both blend ratios occurs. Crosslinked and branched polymer chain topologies are known to exhibit a linear dependency of the shear viscosity to the applied frequency [51]. To confirm this observation, storage moduli (G’) data was plotted against the frequency (Figure A3). The G’ of both blends with SMA tend to reach a plateau for low frequencies around 0.1 rad/s indicating a solid-like behavior [36], due to the graft copolymer formation between SMA and PA66 via imidization reaction. As observed for the binary blends, also with 15 wt% SMA, the 40/60 blend ratio reveals higher viscosities over the full spectrum of frequency.

### 3.2. Blend Morphology

**Figure 4:**
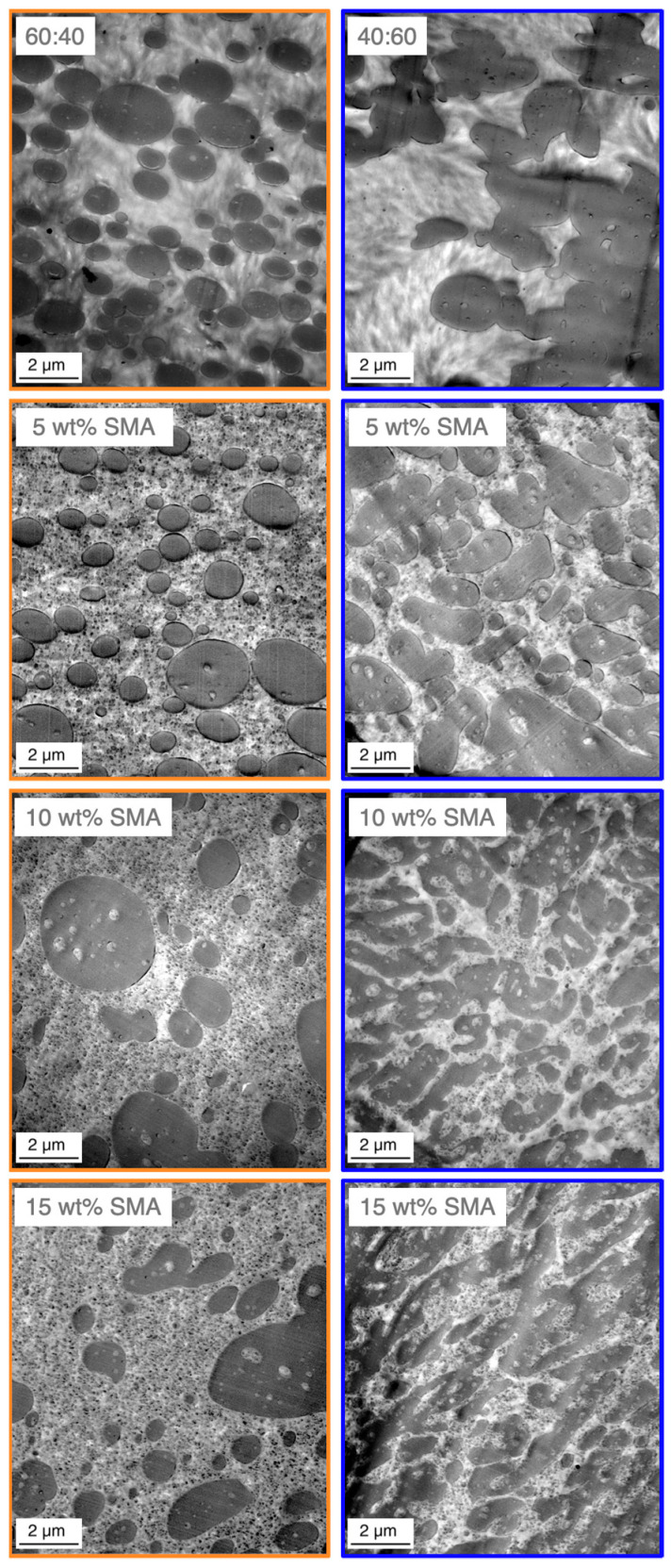
Transmission electron microscopy (TEM) micrographs of SMA compatibilized PA66/PPE ternary blends, for 60/40 (left column) and 40/60 (right column) blend ratio with 0, 5, 10 and 15 wt% SMA.

To gain a deeper understanding about the apparent morphologies, TEM images of the 60/40 and 40/60 blends with SMA contents of 0, 5, 10 and 15 wt% are given in Figure 4. The dark areas represent the PPE phase which was selectively stained prior the microscopy.

Based on the findings from Section 3.1, the PPE forms the dispersed phase as expected, due to its significantly higher viscosity compared to PA66 (Figure A1). According to Figure 4 the 60/40 blend without SMA shows a droplet-sea morphology with distinct ellipsoid-shaped PPE domains in the size range of 200–3200 nm. The PPE domains are not evenly distributed, revealing larger PA-rich areas. Upon addition of SMA, the droplet-sea morphology is maintained, for all SMA contents. With increasing SMA content, the PPE domain sizes shift to a large fraction of small domains in the range of 600 nm together with small fractions of very large domains up to 3500, 4000 and 6500 nm for 5, 10, and 15 wt% SMA content, respectively. For the highest SMA content of 15 wt%, the PPE domains appear elongated and irregularly shaped, indicating a possible beginning of a morphology transition.

Looking at the right column of Figure 4, the 40/60 blends show a different behavior. Without SMA, strong coalescence of the PPE phases occurs. This results in only few distinct PPE domains in the size of 500–2700 nm. The addition of 5 wt% SMA leads to a higher number of distinct PPE domains, proving that SMA acts as a compatibilizing agent and inhibits the coalescence partially. At 10 wt% SMA, a disperse-to-co-continuous transition (DCT), expressed by strongly irregular and elongated domains is observed [52]. A maximum load of 15 wt% SMA represents a complete transition to co-continuity. The filtered PPE domain sizes are summarized in Table 3.

For the 60/40 blends a coarsening of the domain size distribution is explained by the broadened standard deviation with increasing SMA content from 505 to 881 nm. For 40/60 blends, the addition of 5 wt% SMA leads to the formation of slightly smaller PPE domains with a more homogeneous distribution compared to the neat blend. For 10 and 15 wt% SMA contents, the determination of domain sizes is not possible as a morphology transition starts.

A morphology transition is mainly induced either by a change in blend ratio or by a shift of the viscosity ratio between the dispersed and matrix phases [53,54]. For a constant blend ratio, a shift in the morphology induced by SMA copolymers was already reported elsewhere [47]. Inclusions within the PA66 phase, only apparent when SMA was added, leads to an increase of the PA66 viscosity, thus shifting the viscosity ratio between PA66/SMA and PPE to closer values.

The mentioned inclusions appear within the 60/40 and 40/60 blends, regardless of the SMA content (Figure 4). It seems that the SMA’s compatibilizing mechanism is based on an emulsification of the PPE domains, revealing nano-sized droplets by pinch-offs as a result of surface roughening of the PPE phase [14,16,55]. In our previous work [47], we reported that the small droplets either consist of PA66-g-SMA micelles (smaller 50 nm) or more likely PA66-g-SMA micelles swollen by PPE (larger 50 nm). Up to now, no analysis was done to confirm the theory of swollen micelles for PA-based PPE blends. A similar study is only found for PA66/syndiotactic polystyrene blends [14]. To investigate this theory and to understand the effect of blend ratio and SMA content, the domain size distribution of PA66-g-SMA is tabulated in Table 4.

Interestingly, an increase of the micelle sizes is observed for both blend ratios with increasing SMA content, while the standard deviation also increases consistently. In both cases, the average micelle size increases by approximately 30 nm from 5 to 15 wt% SMA content. Additionally, larger domains (up to 18 nm in average) over all considered SMA contents are visible for the PPE-rich blends. As discussed in Section 3.1, a strong interaction between SMA and PPE is evident, which also occurs due to an emulsification process of the PPE when it is melt-blended with PA66 in presence of SMA.

The obtained results allow to support the claimed theory of swollen micelles, as the sizes vary with varying certain parameters, such as blend ratio and SMA content. Both, the PPE content and the SMA content in the blend correlate with the sizes of the micelles, wherein higher PPE and SMA contents enable larger encapsulations of nano-sized PPE in the PA66 phase.

### 3.3. Static Load Behavior (Tensile Mode)

**Figure 5:**
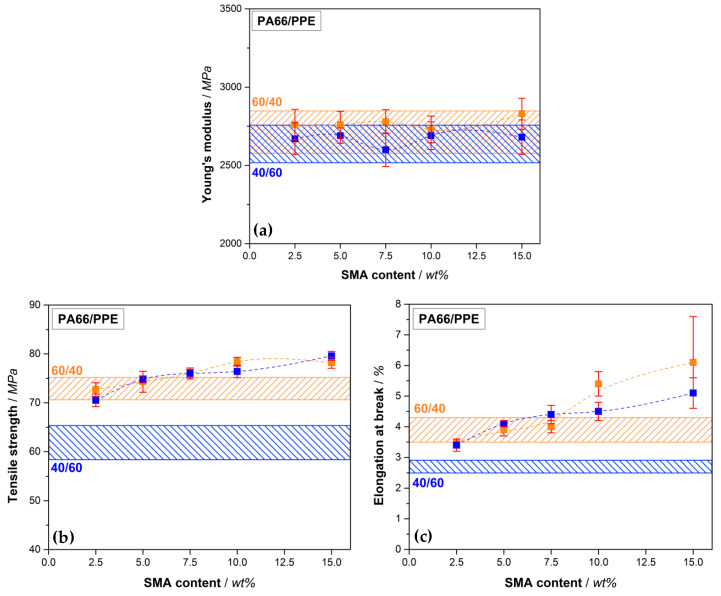
Tensile properties of PA66/PPE/SMA ternary blends with Young’s modulus (**a**), tensile strength (**b**) and elongation at break (**c**), depending on the SMA content, with 60/40 blend ratio (orange rectangular) and 40/60 blend ratio (blue rectangular). Orange (60/40) and blue bars (40/60) stand for the PA66/PPE binary blend values without SMA.

The mechanical properties of PA66/PPE/SMA blends are discussed by considering their response in a tensile test. In Figure 5, the Young’s modulus, tensile strength, and elongation at break are depicted, respectively. The corresponding values are summarized in Table A2.

According to Figure 5a, the Young’s modulus of the 60/40 blends give rise to slightly higher values compared to the 40/60 blends, yet all values are considered comparable as no significant difference is recognizable. The modulus is highly influenced by materials with dissimilar moduli of the individual components, such as polymer (low modulus) and glass fibers (very high modulus) [54]. As the moduli of all blend components range between 2400 to 3000 MPa, the resulting mixed modulus will not differ significantly at different ratios. In regard to tensile strength (Figure 5b), a similar trend is seen. The PA66-rich blends (60/40) result in overall higher values. A marginal increase of the values is observed with SMA contents up to 5 wt%. For 10 and 15 wt% SMA content a significant increase in tensile strength is reached to 78.4 (+7.7%) and 78.2 MPa (+7.4%), respectively. As the morphology remains a droplet-sea structure for all SMA contents, the improvement can be assigned to the effect of SMA and cannot be caused by a change in morphology. For the 40/60 blends a drastic increase in tensile strength from 61.8 to 70.5 MPa (+14.1%) is apparent already with only 2.5 wt% SMA content. Further increase in SMA content (5 wt%) reveals further enhancement of the tensile strength (+21.0%) without a significant morphology change as seen in Figure 4. As mentioned, the strong affinity of SMA to PPE results in stronger interactions when a sufficient amount of PPE is given. After passing a plateau at 10 wt% SMA, the maximum tensile strength of 79.6 MPa (+28.8%) is achieved at 15 wt% SMA content, which is the highest obtained value within this comparison. Between 5 and 15 wt% the blend passes the DCT to form bi-continuous structures at 15 wt% as seen in Figure 4. Apparently, a morphology transition suppresses the compatibilizing efficiency of the SMA, resulting in a slight or no improvement of the tensile strength. A further increase in tensile strength after the mentioned plateau for 15 wt% SMA is in good agreement with this hypothesis.

For 40/60 blends, the same trend is also seen for 2.5 and 5 wt% SMA in elongation at break values (Figure 5c). A significant growth in the values (+25.9% and +51.9%) is followed by a plateau up to 10 wt% SMA (+66.7%). For 15 wt% SMA the value again rises to 5.1% resulting an improvement of 88.9% compared to the neat 40/60 blend. For the 60/40 blends, a significant increase in elongation at break first starts at 10 wt% SMA (+38.5%) reaching its maximum at 15 wt% SMA with 6.1% (+56.4%). However, the higher value goes hand in hand with a large standard deviation. Here, we assume that defects may cause premature cracking of the samples, resulting in unexpectedly high deviations within the material.

From the tensile properties it can be concluded that the 60/40 blends yield materials with the highest absolute tensile values. Nevertheless, the SMA reveals a higher efficiency when a higher PPE content is available (40/60 blends), showing the greatest relative improvement compared to the binary blend without SMA. Comparing the findings with available literature, also stronger interactions, thus higher relative improvements are found with higher amounts of PPE [34].

#### Fracture Analysis

**Figure 6:**
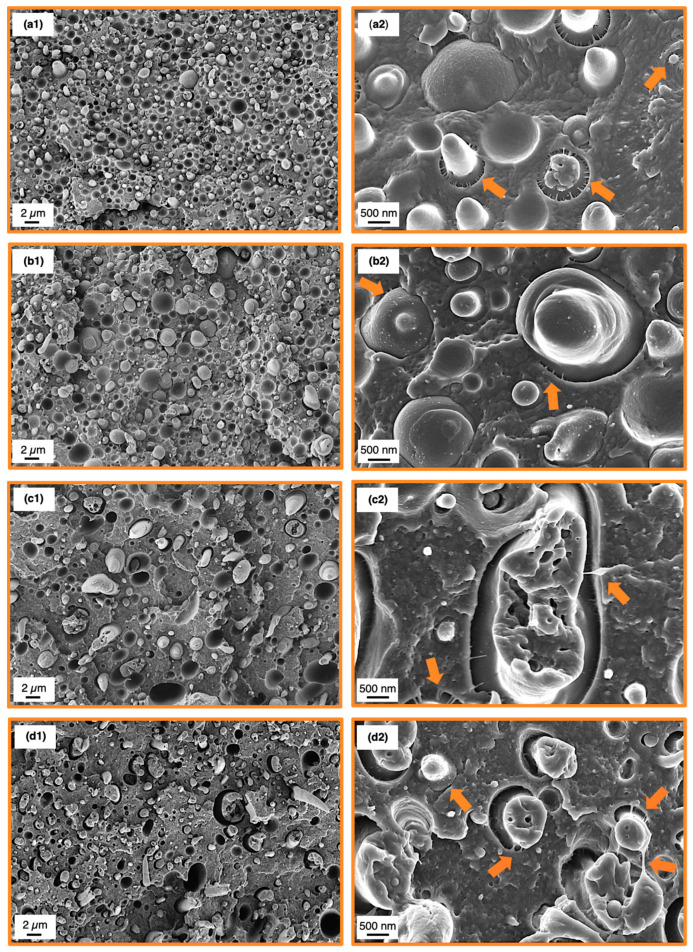
Scanning electron microscopy (SEM) micrographs subsequent to tensile testing of the 60/40 binary blend (**a1**), 5 wt% SMA (**b1**), 10 wt% SMA (c1) and 15 wt% SMA (d1). Higher magnifications of the corresponding blends are depicted at the right column (**a2**,**b2**,**c2**,**d2**). Orange arrows indicate matrix fibrillations.

**Figure 7:**
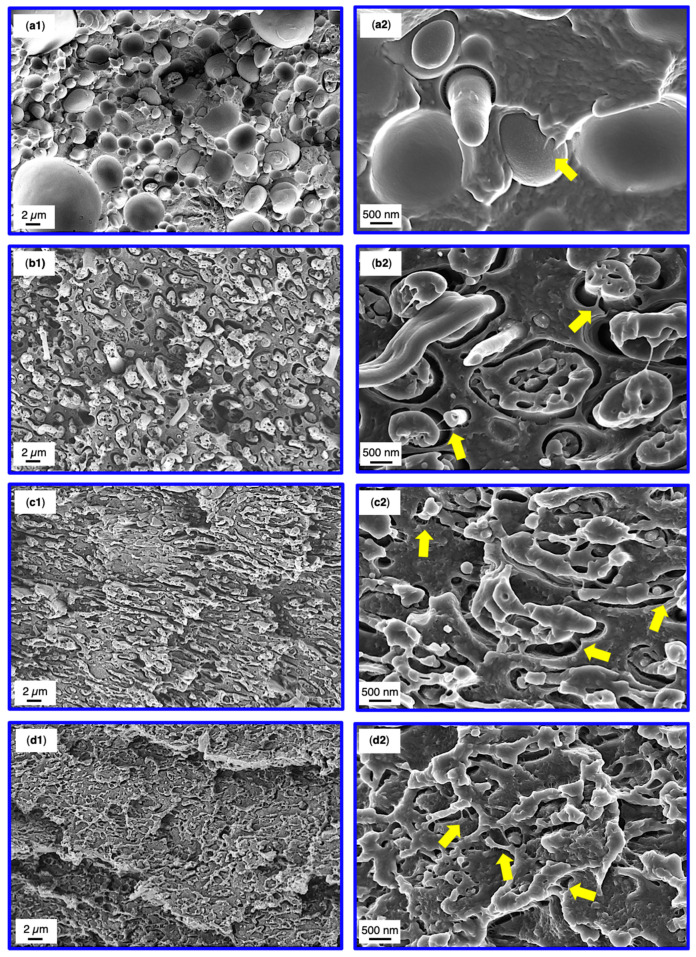
SEM micrographs subsequent to tensile testing of the 40/60 binary blend (**a1**), 5 wt% SMA (**b1**), 10 wt% SMA (**c1**) and 15 wt% SMA (d1). Higher magnifications of the corresponding blends are depicted at the right column (**a2**,**b2**,**c2**,**d2**). Yellow arrows indicate matrix fibrillations.

SEM analysis of the SMA compatibilized blends was conducted after tensile test to correlate the results with the corresponding fracture surfaces. The fractographs of 60/40 blends are depicted in Figure 6, whereas the 40/60 fracture surfaces are shown in Figure 7. Overview graphs with 2500× magnification are placed in the left column (Figure 6 and Figure 7(a1–d1)) together with graphs at a magnification of 15,000× for the analysis of the micromechanics (Figure 6 and Figure 7(a2–d2)).

Starting with the 60/40 blends, the reference blend without SMA (Figure 6(a1,a2)) shows a rather featureless fracture surface with weakly expressed crack deflections. As all samples were dried prior testing, the PA66 phase is in a brittle state, thus not showing any plastic deformation. Pull-outs of PPE domains are clearly the main deformation mechanism indicating a weak interfacial bonding between PA66 and PPE. Nevertheless, PPE domains with low plastic deformation are apparent sporadically, which is due to existing bonds between PA66 and PPE (Figure 6(a2)). These bonds are emphasized by local fibrillations of the matrix at the interface (orange arrows) [56,57] to ease energy dissipation enabled by debonding and mentioned fibrillation. A sufficient amount of PA66 enables covalent bonding of PA66 and PPE, as latter consists of terminal hydroxyl moieties [58,59]. The mentioned hydroxyl groups will react with carboxyl moieties of the PA66 under esterification to form predominantly linearly extended diblock copolymers (PA66-b-PPE) [60].

A similar behavior is seen with the addition of 5 wt% SMA (Figure 6(b1,b2)). The fracture surface appears rough, having stronger crack deflections compared to the neat blend. Interestingly, the elongation of PPE domains is less frequent. An elongation of PPE domains only occurs together with matrix fibrillations (valid for droplet-sea morphology) indicating a strong bond, which is almost absent for 5 wt% SMA. A reduced interfacial strength combined with stronger crack deflections give rise to tensile values approaching the values of the reference blend. As SMA has a strong interfacial activity, it inhibits the formation of PA66-b-PPE copolymers at the interface as it occupies these areas. For small amounts of SMA, the interfaces are disordered and may even reveal deteriorated properties [44]. Further addition of SMA to 10 and 15 wt% intensifies the step-like crack deflections resulting in a rough surface with a decreasing amount of PPE pull-outs. The PPE domains appear more elongated caused by strong interfacial bonds, enabled by the SMA. After a certain extent of elongation, cohesive failure of the PPE domains becomes predominant. Interparticle bridging of PPE (Figure 6(d2)) (orange arrow, bottom right)) is a clear indication of the strong interfacial bonding between the blend components. The graphs at higher magnification also depict the nano-micelles described in Section 3.2, which become more noticeable with increasing SMA content (bright spots in Figure 6(b2–d2)). These micelles also undergo a plastic deformation in the same manner as the larger PPE phases, corroborating our theory of that the PA66-g-SMA micelles are swollen by PPE.

Looking at the 40/60 blends (Figure 7), the binary blend indicates a rather rough fracture surface due to the broad size distribution of the PPE domains. Occasionally, PPE debonding under elongation, coexisting with matrix fibrillations is found (Figure 7, yellow arrow). The fracture mechanism is dominated by pull-outs of the PPE represented by smooth domain surfaces and cavities. Upon addition of SMA, a morphology change is apparent (as discussed in Section 3.2) resulting in very thin PPE structures, which are hardly visible at a magnification of 2500× (Figure 7(d1)). The sizes of mentioned structures match very well with the observed dimensions in Figure 4. With an increasing SMA content, step-like crack deflections become more pronounced, absorbing the majority of the applied force. For 5 wt% SMA the droplet-sea morphology still exists. This reveals a strong elongation of the PPE domains with elongation ratios up to 5-times the initial domain diameter. The higher rate of elongation must be enabled by a stronger interfacial bonding. The debonding and elongation of PPE, together with matrix fibrillations and interparticle bridging represent the main micro-mechanical fracture mechanisms. Such strong interactions of SMA and PPE were not observed for the 60/40 blends, supporting the discussion so far.

For 10 and 15 wt% SMA, the elongation rate of the PPE phase is not as prominent as for 5 wt% SMA. Yet, the co-continuous structures enable a mechanical interlocking (acting as mechanical anchors) of the blend partners leading to very pronounced matrix deflections with high energy absorption. The partially elongated PPE phases experience cohesive failure thereafter, having strong matrix fibrillations (Figure 7(c2,d2), yellow arrows). As seen for the 60/40 blends with SMA, the emulsified PPE nano domains are apparent in Figure 7(b2–d2)), although not as visible as for 60/40, due to the agitated fracture surfaces.

It can be concluded that the 60/40 blends reveal higher energy dissipation due to a higher amount of PA66, resulting in higher absolute tensile values compared to 40/60 blends. The failure mechanisms are pull-outs and debonding of PPE coexisting with moderate crack deflection. For the 40/60 blends, stronger interactions of SMA with PPE is seen, where debonding and elongation of PPE is existent. A change in morphology combined with the stronger interfacial bonding and nano-emulsification of PPE enable even higher tensile values.

## 4. Conclusions

Within this study, we described how a SMA copolymer can effectively compatibilize PA66/PPE blends. Structure-property correlations were successfully established, linking the effect of SMA content and the PA66/PPE blend ratio. It was shown that the SMA is completely miscible with PPE. It has strong interfacial interactions when it is blended with PA66. Regardless of the blend ratio, an emulsification of PPE for all blends containing SMA was found. The diameter range of the emulsified PPE phase was 60 to 160 nm. The emulsification was possible due to surface roughening of the PPE domains in the presence of SMA, leading to pinch-offs of PPE surrounded by SMA-g-PA66. With higher amount of PPE in the 40/60 blends, the size of the emulsified PPE domains increased. With this behavior we were able to justify our hypothesis of PPE being encapsulated within PA66-g-SMA micelles. Simultaneously, a morphology transition was found with higher amounts of SMA (10 and 15 wt%) due to the strong increase in the viscosity of PA66. For 60/40, the effect of the blend ratio is overwhelming in terms of the viscosity increase of the PA66. This does not allow for a morphology change, thus all blends remained as a droplet-sea structure. In regard to tensile properties, the blend ratio of 60/40 appeared beneficial revealing the highest values in Young’s modulus and elongation at break in the presence of 15 wt% SMA content. However, only a slight improvement in tensile strength (+7.7%) was possible with 60/40 ratio and 10 wt% SMA. In contrast, drastic improvements for 40/60 blends were found upon the addition of SMA, where tensile strength and elongation at break increased by 28.8% and 88.9%, respectively. This trend was also described elsewhere [37] for PA6/PPE blends. Noticeable improvements in tensile properties were also achieved when PPE was the majority phase. The reinforcement of the blend was explained by a strong interfacial bonding between PA66 and PPE enabled by SMA. PPE debonding and elongation with strong matrix fibrillation were identified as driving forces on micro-mechanical scale.

Since characterization values all refer to the dry state of blends, we aim to investigate the effect of humidity on the mechanical properties in future. Here, we expect a brittle to ductile transition of the PA66, possibly leading to improved stress transfer at the blend interfaces.

## Figures and Tables

**Table 1:** Properties of blend components.

Polymer	Molecular Weight (M_w_) [g/mol]	Polydispersity [a.u.]	Maleic Anhydride (MA) Concentration [wt%]	Supplier
PA66	60,000	1.74	-	BASF SE (Ludwigshafen, Germany)
PPE	36,800	1.98	-	Asahi Kasei K.K. (Chiyoda, Japan)
SMA	245,000 ^a^	-	8 ^a^	Polyscope B.V. (Geleen, The Netherlands)

^a^ Technical data sheet values [48].

**Table 2:** Glass transition temperatures (T_g_) of SMA and poly (2,6-dimethyl-1,4-phenylene ether) (PPE) (tan δ maximum) for both references (60/40 and 40/60 PA66/PPE) and 15 wt% SMA content.

Sample	Glass Transition Temperature (T_g_) SMA	T_g_ PPE
60/40 PA66/PPE	-	217.2
+15 wt% SMA	138.3	210.0
40/60 PA66/PPE	-	217.4
+15 wt% SMA	135.0	210.0

**Table 3:** Distribution analysis of PPE domain sizes, considering transmission electron microscopy (TEM) micrographs as seen in Figure 4, with a size filter between 200–3000 nm.

PA66/PPE	60/40 [nm]	40/60 [nm]
Neat	1077 ± 505	1629 ± 1148
5 wt% SMA	937 ± 602	1345 ± 669
10 wt% SMA	1063 ± 750	transition region
15 wt% SMA	1105 ± 881	

**Table 4:** Size distribution analysis of swollen Styrene-maleic anhydride copolymer-graft-polyamide 66 (SMA-g-PA66) micelles found in the PA66 phase, determined from the TEM micrographs in Figure 4, with a size filter between 0–200 nm.

PA66/PPE	60/40 [nm]	40/60 [nm]
Neat	not applicable	
5 wt% SMA	80 ± 20	94 ± 28
10 wt% SMA	85 ± 26	95 ± 34
15 wt% SMA	105 ± 37	123 ± 39

## Appendix A

**Table A1:** Property overview of non-reinforced and 30 wt% glass fiber (GF) reinforced polyamide 6 (PA6) and PA66. Reproduced from [61].

Properties	PA6	PA6 GF30	PA66	PA66 GF30
Water absorption@23 °C [%]	9.5	6.5	8.5	5.5
Moisture absorption @23 °C/50% rel. humidity [%]	3.0	2.1	2.8	1.7
Youngs modulus [MPa]	3200	9800	3600	10,500
Charpy Notched Impact @23 °C [kJ/m^2^]	10	12	12	10
HDT-A/1.8 MPa	55	200	75	240
HDT-B/0.45 MPa	160	215	215	250
Crystallization rate (=Cooling/Cycle time)	Slower (longer cooling/ cycle time)		Faster (shorter cooling/ cycle time)	

**Table A2:** Summary of the tensile performance for the PA66/PPE/SMA ternary blends.

PA66/PPE	Youngs Modulus [MPa]		Tensile Strength [MPa]		Elongation at Break [%]	
	60/40	40/60	60/40	40/60	60/40	40/60
Neat	2710 ± 135	2640 ± 114	72.8 ± 2.1	61.8 ± 3.3	3.9 ± 0.4	2.7 ± 0.2
2.5 wt% SMA	2760 ± 98	2670 ± 101	72.7 ± 1.4	70.5 ± 1.3	3.5 ± 0.1	3.4 ± 0.2
5 wt% SMA	2760 ± 85	2690 ± 49	74.3 ± 2.1	74.8 ± 0.7	3.9 ± 0.2	4.1 ± 0.1
7.5 wt% SMA	2780 ± 76	2600 ± 107	76.0 ± 0.8	76.0 ± 1.1	4.0 ± 0.2	4.4 ± 0.3
10 wt% SMA	2730 ± 85	2690 ± 88	78.4 ± 0.9	76.4 ± 1.3	5.4 ± 0.4	4.5 ± 0.3
15 wt% SMA	2830 ± 99	2680 ± 110	78.2 ± 1.1	79.6 ± 0.9	6.1 ± 1.5	5.1 ± 0.5
